# Does South Korea have hidden female smokers: discrepancies in smoking rates between self-reports and urinary cotinine level

**DOI:** 10.1186/s12905-014-0156-z

**Published:** 2014-12-12

**Authors:** Myung Bae Park, Chun-Bae Kim, Eun Woo Nam, Kyeong Soo Hong

**Affiliations:** Department of Health Administration, Yonsei University, Gangwon-Do, Republic of Korea; Department of Preventive Medicine, Yonsei University Wonju College of Medicine, 162 Ilsan-Dong, Wonju-City, Gangwon-Do 220-701 Republic of Korea; Yonsei University Institute for Poverty Alleviation and International Development, Gangwon-Do, Republic of Korea; Healthy City Research Center, Institute of Health and Welfare, Yonsei University, Gangwon-Do, Republic of Korea; Korea Health Promotion Foundation, Seoul, Republic of Korea

**Keywords:** Female smokers, Self-reported surveys, South Korea, Urinary cotinine concentration

## Abstract

**Background:**

Female smoking is perceived very negatively in East Asian countries such as South Korea, Japan, and China, as well as in Islamic countries. These countries’ self-reported surveys (SRs) tend to produce results that underestimate the number of smokers, owing to the social desirability response bias. The present study seeks to assess South Korea, Europe, and the Americas, by comparing data from SRs with those from urinary cotinine samples.

**Methods:**

Current smoking rates were calculated using the SRs and the urinary cotinine concentration (UCC) methods according to socioeconomic factors. In order to examine response accuracy regarding current smoking status in the SRs, participants who both completed the SRs and acquired UCC results were subject to analyses of sensitivity, specificity, positive predictive value (PPV), negative predictive value (NPV), and difference ratio (DR) with respect to gender, age, region, economic level, household status, and the presence of chronic disease.

**Results:**

Based on self-reports, the current smoking rate among women was 7.1% (official smoking rates), while that according to the UCC was 18.2%; the rates for men were 47.8% and 55.1%, respectively. The sensitivity of males was 0.8553, the specificity 0.9768, PPV 0.9783, NPV 0.8465, and the difference ratio (DR) was 1.143. The sensitivity for females was 0.3670, the specificity 0.9956, PPV 0.9486, NPV 0.8761, and the DR was 2.6. These results exhibit a very low response alignment rate compared to males.

**Conclusion:**

This study shows that the actual female smoking rate is significantly higher than that reported officially, but also that the gap is decreasing steadily. Females exhibited a higher rate of false responses, which resulted in an underestimation of the female smoking rate.

## Background

In most countries, the smoking rate for women is lower than that for men. This gender difference indicates regional and cultural variations in Africa, the Asia-Pacific region, and Latin America [1]. Much greater gender differences were found in East and Southeast Asian in countries such as South Korea, Indonesia, and China, compared to Europe and the U.S. [2].

In South Korea, the smoking rate of adults over the age of 19 was 79.3% for males and 12.6% for females, in 1980. However, both an anti-smoking campaign initiated by citizen activists in the late 1980s, and the introduction of the Law for the Promotion of the Nation’s Health that regulated smoking in 1995 have affected South Korea’s smoking rates, which have been steadily decreasing. In 2007, the male smoking rate was 45.0% and the female smoking rate was 5.3%. Since then, the rates of smoking have remained more or less constant: those of men and women were 47.3% and 6.8%, respectively, in 2011 [3]. South Korea has the highest male smoking rate of all countries in the Organization for Economic Cooperation and Development (OECD), but the lowest female smoking rate [4].

Studies on the rates of smoking are generally conducted through self-reported surveys (SRs) because of their convenience and economic feasibility. However, SRs tend to produce results that underestimate the number of smokers, owing to social desirability response bias [5-7]. Furthermore, smoking among women is regarded more negatively in South Korea than in Western countries. For these reasons, South Korea’s actual female smoking rate is also thought to be higher than what has been officially indicated [8]. Consequently, South Korea’s distinction of having the lowest female smoking rate of all OECD countries (OECD’s smoking rate includes participants aged 15 and above; http://stats.oecd.org/Index.aspx) is subject to doubt.

The citizens’ activist group called the Korean Association on Smoking and Health began investigating South Korea’s smoking rates in 1980. In 1998, the Korea Centers for Disease Control and Prevention (KCDC) began investigating the smoking rate. Although comparative studies have been conducted on regional and specific demographic levels (including hospital patients) using biological markers to determine the accuracy of SRs, very few studies estimated the real smoking rates using the national index [9], the Korean National Health and Nutrition Examination Survey (KNHANEs). Biological markers that measure smoking include cotinine, nicotine, carbon monoxide, thiocyanate, and carboxy hemoglobin. Among these, cotinine is a metabolite of nicotine that has a relatively long half-life compared to other markers, making it a reliable indicator when used to identify smokers [10-12].

The aims of this study were, first, to compare smoking rates based on self-reported survey results and urinary cotinine concentration (UCC) test results, and second, to estimate actual smoking rates by year using a biological marker. It is the first study to analyze the annual trends of actual smoking rates in South Korea by reflecting the results of biomarker tests.

## Methods

### Study population

The KNHANEs 20 households were selected from 192 areas by probability sampling. The primary sampling unit was administrative unit (i.e., eup-myeon-dong), the secondary sampling was done by census, and the tertiary sampling was done at the household level. All members of the selected households were included as survey participants. This survey comprises three areas: physical examinations, health-related surveys, and nutrition surveys. During the study period, examiners visited different areas using a mobile examination vehicle and conducted check-ups that included blood and urine tests, X-rays, and oral examinations. The examiners also conducted a self-reported health survey that posed questions regarding health behaviors, clinic use, and quality of life. One week following the check-ups and health surveys, a nutritional survey was conducted through a home visitation. In principle, all participants were investigated in all three areas. The SRs were carried out during face-to-face interviews.

Data were gathered from a study population of 26,593 adults over 19 years of age who responded to the question regarding smoking on the KNHANEs between 2008 and 2011. From the above population, 14,086 respondents were selected for further analysis, because their urinary cotinine data were made available for use. The survey was approved by the Institutional Review Board (IRB) of the KCDC (approval nos. 2008-04EXP-01-C, 2009-01CON-03-2C, 2010-02CON-21-C, 2011-02CON-06-C). The KNHANEs has a stratified multistage sampling design that examines all household members above the age of one.

### Measures

The current smoking rate is based on the definition of a smoker as someone who has smoked at least 100 individual cigarettes in his or her lifetime and who, at the time of interview, reported smoking every day or some days. Measurements of UCC are taken using gas chromatography–mass spectrometry, specifically with the detector model Clarus 600 T of Finland PerkinElmer. The indicators used in the measurement are cotinine and diphenylamine (Aldrich, USA). The UCC threshold for identifying smokers is generally set at 20–100 ng/ml [7,13,14], but the Society for Research on Nicotine and Tobacco has suggested that the standardized threshold of urinary cotinine be set at 50 ng/ml [15]. It should be noted that this suggestion for standardization reflects a Western model that does not necessarily apply to the characteristics of South Korea or other Asian countries. In South Korea, for instance, studies involving interviews and longitudinal observations of certain populations have suggested UCC thresholds as low as 20 ng/ml can indicate smokers [9]. Admittedly, however, such a low threshold is not sensitive to the detection of cotinine owing to unrelated factors such as secondhand smoking. Therefore, the present research used one of the generally accepted threshold levels of UCC ≥ 30 ng/ml [16].

### Data analysis

The KNHANEs is a stratified multistage sampling design. In all calculations, individual weighted factors were used according to the KCDC guidelines. In addition, integrated weights were applied due to the merged years. The current smoking rate, according to the official national data collected from SRs, and the current smoking rate, according to the UCC data, were analyzed using socioeconomic variables.

In order to examine response accuracy regarding current smoking status in the SRs, participants in both the SRs and the UCC data were subject to analyses of sensitivity, specificity, positive predictive value (PPV), negative predictive value (NPV), and difference ratio (DR) with respect to characteristics including gender, age, region, economic level, household status, and the presence of chronic disease. Annual trends in smoking rates were estimated using the frequentist probability and Bayesian Theory. Igor et al. [17] suggested using the Bayesian Theory in the estimation of smoking rates. The calculation of that is composed of r0 (SRs non-smokers/UCC non-smokers), r1 (SRs smokers/UCC smokers), true positives (TP), false negatives (FN), true negatives (TN), and false positives (FP). The formula is “Prevalence = (r1*TP + r0*FN)/(r1*TP + r0*TN + r0*FN + r1*FP).” Statistical analysis was performed using SAS 9.3 for Windows.

## Results

The current smoking rate of adult males according to the SRs is 47.8%, while the current smoking rate according to the UCC is 55.1%. The smoking rate among males was 55.1% according to the UCC, which is 7.3% higher than the official smoking rates. In particular, the female smoking rate according to the UCC (18.2%) was more than twice as high as the official smoking rate indicated by the SRs (7.1%).

Smoking rates were highest among males in their 30s and females in their 20s, and in general, the younger age groups (20s and 30s) exhibited higher overall smoking rates. By age group, the highest smoking rates were observed in the 70+ groups for both males and females. The widest gaps between the two indicators were found in the 70+ group for males, and in the 20–29 group for females. By household, one-person households showed the highest smoking rates for both males and females, but the gap was wider in households with two persons or more. By individual income quartile, the “low” group showed the highest smoking rates for both males and females. By region, “rural” regions showed the highest smoking rates among males, along with the widest inter-indicator gap. For females, “other urban city” showed the highest smoking rates and the widest inter-indicator gap. By cancer incidence experience, the experience group showed the highest smoking rates for both males and females. For males, the gap was wider among those who reported cancer incidence experience, while for females it was wider among those without cancer (see Table 1).

**Table 1:** **The difference between smoking rates according to SRs and UCC (2008–2011)**

									Unit: n*, %**	
	**Male**					**Female**				
	**SRs (A)** **N**_1_ ** = 11,353**	**95% CI**	**UCC (B)** **n**_1_ ** = 6,618**	**95% CI**	**B-A**	**SRs (C)** **N**_2_ ** = 15,240**	**95% CI**	**UCC (D)** **n**_2_ ** = 7,468**	**95% CI**	**D-C**
**Overall**	4,909 (47.8)	(45.6-48.9)	3,442 (55.1)	(53.6-55.6)	7.3	920 (7.1)	(6.5-7.6)	1,283 (18.2)	(16.8-19.6)	11.1
**Age (years)**										
20 - 29	710 (50.2)	(47.3-53.1)	586 (56.5)	(53.2-59.9)	6.3	216 (11.4)	(9.5-13.2)	264 (24.9)	(21.7-28.1)	13.5
30 - 39	1,262 (59.8)	(57.5-62.1)	871 (64.7)	(61.9-67.5)	4.9	213 (8.4)	(7.3-9.6)	305 (21.8)	(19.3-24.3)	13.4
40 - 49	1,094 (49.9)	(47.4-52.4)	774 (56.6)	(53.5-59.6)	6.7	150 (5.8)	(4.8-6.8)	247 (17.5)	(15.0-19.9)	11.7
50 - 59	847 (43.7)	(41.1-46.4)	574 (49.7)	(46.5-52.9)	6.0	107 (4.7)	(3.6-5.8)	182 (13.2)	(11.0-15.4)	8.5
60 - 69	601 (33.0)	(30.3-35.7)	408 (41.5)	(37.7-45.3)	8.5	99 (4.0)	(3.1-5.0)	150 (10.7)	(8.5-12.8)	6.7
70 over	395 (26.2)	(23.4-28.9)	229 (38.3)	(33.2-43.4)	12.1	135 (6.3)	(5.0-7.5)	135 (15.6)	(12.6-18.7)	9.3
**Number of household**										
1	273 (56.1)	(51.1-61.0)	186 (58.5)	(52.6-64.4)	2.4	137 (11.9)	(9.5-14.2)	121 (21.2)	(17.4-25.0)	9.3
2 over	4,636 (47.3)	(46.2-48.5)	3,256 (54.9)	(53.3-56.4)	7.6	783 (6.7)	(6.2-7.3)	1,162 (18.0)	(16.6-19.4)	11.3
**Income quartile**										
Low	1,355 (53.8)	(51.5-56.2)	924 (60.3)	(57.3-63.2)	6.5	331 (10.5)	(9.2-11.8)	378 (21.4)	(19.0-23.8)	10.9
Middle low	1,272 (48.8)	(46.6-51.1)	902 (57.1)	(54.1-60.1)	8.3	242 (7.1)	(6.1-8.1)	324 (17.9)	(15.7-20.0)	10.8
Middle high	1,143 (46.4)	(44.1-48.7)	780 (53.5)	(50.5-56.4)	7.1	177 (5.7)	(4.6-6.7)	252 (15.4)	(13.1-17.7)	9.7
High	1,063 (41.8)	(39.6-44.1)	770 (49.2)	(46.3-52.1)	7.4	152 (4.9)	(3.9-5.8)	300 (17.8)	(15.3-20.4)	12.9
**Residential area**										
Metro	2,152 (47.0)	(45.3-48.7)	1,487 (52.9)	(50.6-55.2)	5.9	418 (6.8)	(6.1-7.6)	563 (17.4)	(15.5-19.4)	10.6
Other urban city	1,697 (48.2)	(46.2-50.1)	1,224 (56.6)	(54.3-58.8)	8.4	338 (7.9)	(6.8-8.9)	460 (20.0)	(17.7-22.3)	12.1
Rural area	1,060 (49.1)	(46.5-51.7)	1,402 (58.0)	(54.1-61.9)	8.9	164 (6.2)	(4.9-7.5)	260 (16.8)	(13.8-19.9)	10.6
**NCD** ***										
Yes	1,066 (37.9)	(35.7-40.2)	744 (46.8)	(43.8-49.8)	8.9	184 (4.6)	(3.8-5.3)	250 (12.8)	(10.8-14.8)	8.2
No	3,843 (50.6)	(49.2-51.9)	2,698 (57.2)	(55.5-58.9)	6.6	736 (7.8)	(7.1-8.5)	1,033 (19.7)	(18.1-21.2)	11.9
**Cancer**										
Yes	54 (20.5)	(14.2-26.7)	46 (38.0)	(28.4-47.5)	17.5	896 (3.6)	(1.7-5.5)	29 (11.1)	(6.5-15.8)	7.5
No	4,855 (48.3)	(47.1-49.4)	3,396 (55.3)	(53.8-56.8)	7.0	24 (7.4)	(6.6-7.8)	1,254 (18.4)	(17.0-19.8)	11.0

SRs, Self-reported surveys; UCC, Urinary cotinine concentration.

*Unweighted number of respondents. **Weighted percentage.

***NCD: Non-Communicable Disease (physician diagnosis about hypertension, diabetes mellitus, dyslipidemia, etc.).

Among the SR respondents, there were 14,086 individuals who participated in the urinary cotinine measurements. After four males and five females who answered “I don’t know” to the question of current smoking status were excluded, data from a total of 6,614 males and 7,463 females were analyzed to determine the response accuracy of the SRs. The sensitivity of males was 0.8553, specificity 0.9768, PPV 0.9783, NPV 0.8465, and DR was 1.143. The sensitivity for females was 0.3670, specificity 0.9956, PPV 0.9486, NPV 0.8761, and DR was 2.6. These results exhibit a very low response alignment rate compared to males. In the case of females, the sensitivity was highest among female one-person households (0.5466). Women in other age groups did not exhibit a sensitivity value that exceeded 0.5. With respect to the number of household members, the sensitivity value of a minimum two-person household was 0.3530, and DR was 2.687. These results exhibit a response alignment lower than that of a one-person household. According to the sensitivity values with respect to income quartiles, the “low” quartile had a value of 0.4581, “middle low” 0.4080, “middle high” 0.2883, and “high” 0.2883. As income level increased, the response alignment decreased (see Table 2).

**Table 2:** **Summarizing results of the accuracy for smoking rates according to UCC and SRs**

	**Male (n**_1_* **= 6,614)**					**Female (n**_2_* **= 7,463)**				
	SN**	SP**	PPV**	NPV**	DR**	SN**	SP**	PPV**	NPV**	DR**
**Overall (95% CI)**	0.8553 (.8550-.8556)	0.9768 (.9766-.9799)	0.9783 (.9782-.9785)	0.8465 (.8463-.8468)	1.143	0.3670 (.3663-.3678)	0.9956 (.9955-.9966)	0.9486 (.9480-.9491)	0.8761 (.8759-.8763)	2.600
**Age (years)**										
20 – 29	0.8585 (.8580-.8591)	0.9819 (.9816-9821)	0.9840 (.9838-.9842)	0.8422 (.8416-.8428)	1.146	0.4149 (.4135-.4163)	0.9867 (.9865-.9869)	0.9119 (.9107-.9131)	0.8358 (.8353-.8464)	2.204
30 – 39	0.8804 (.8799-.8809)	0.9366 (.9361-9371)	0.9622 (.9619-.9622)	0.8104 (.8097-.8112)	1.093	0.3733 (.3718-.3748).	0.9969 (.9969-9970)	0.9715 (.9707-.9723)	0.8510 (.8504-.8515)	2.595
40 – 49	0.8624 (.8618-.8629)	0.9812 (.9809-9814)	0.9835 (.9833-.9837)	0.8455 (.8449-.8461)	1.141	0.3125 (.3110-.3140)	0.9993 (.9992-.9993)	0.9888 (.9882-.9895)	0.8727 (.8722-.8733)	3.182
50 – 59	0.8393 (.8385-.8400)	0.9879 (.9877-9882)	0.9856 (.9854-.9859)	0.8618 (.8611-.8624)	1.175	0.2969 (.2950-.2988)	0.9964 (.9963-9965)	0.9262 (.9243-.9281)	0.9027 (.9022-.9031)	3.143
60 – 69	0.8087 (.8075-.8099)	0.9915 (.9913-.9917)	0.9854 (.9850-.9858)	0.8798 (.8790-.8806)	1.221	0.3649 (.3621-.3677)	0.9968 (.9967-.9970)	0.9316 (.9293-.9339)	0.9302 (.9297-9307)	2.610
70 over	0.7482 (.7462-.7501)	0.9959 (.9957-.9962)	0.9913 (.9909-.9918)	0.8643 (.8631-.8654)	1.325	0.4484 (.4454-.4513)	1.0000	1.0000	0.9071 (.9064-.9078)	2.229
**Number of household**										
1	0.9146 (.9137-.9154)	0.9830 (.9825-9835)	0.9870 (.9866-.9873)	.8908 (.8897-.8919)	1.079	0.5466 (.5438-.5494)	0.9960 (.9960-.9963)	1.0000	0.8908 (.8900-.8917)	1.782
2 over	0.8545 (.8539-.8552)	0.9764 (.9763-.9765)	0.9777 (.9776-.9778)	.8448 (.8445-.8551)	1.149	0.3530 (.3522-.3537)	0.9955 (.9955-.9956)	0.9455 (.9449-.9461)	0.8752 (.8750-.8754)	2.687
**Income quartile**										
Low	0.8893 (.8888-8898)	0.9784 (.9781-.9786)	0.9842 (.9840-.9844)	0.8536 (.8530-.8542)	1.108	0.4581 (.4567-4595)	0.9944 (.9943-.9945)	0.9568 (.9560-.9576)	0.8712 (.8707-.8816)	2.098
Middle low	0.8364 (.8359-.8370)	0.9817 (.9814-.9819)	0.9838 (.9836-.9840)	0.8184 (.8177-.8190)	1.175	0.4080 (.4065-4095)	0.9964 (.9963-.9965)	0.9611 (.9602-.9620)	0.8856 (.8852-.8860)	2.355
Middle high	0.8651 (.8646-.8657)	0.9712 (.9709-9715)	0.9719 (.9716-.9721)	0.8624 (.8618-.8624)	1.124	0.2883 (.2868-2899)	0.9937 (.9936-.9938)	0.8923 (.8905-.8942)	0.8848 (.8843-.8852)	3.080
High	0.8275 (.8269-.8282)	0.9750 (.9747-.9752)	0.9698 (.9694-.9701)	0.8537 (.8531-.8542)	1.171	0.2746 (.2732-2759)	0.9974 (.9974-.9975)	0.9586 (.9575-.9598)	0.8636 (.8631-.8640)	3.490
**Residential area**										
Metro	0.8576 (.8572-.8580)	0.9731 (.9729-.9733)	0.9728 (.9726-.9730)	0.8587 (.8583-.8591)	1.133	0.3727 (.3717-.3738)	0.9945 (.9944-9946)	0.9348 (.9339-.9356)	0.8826 (.8823-.8829)	2.522
Other urban city	0.8550 (.8546-.8555)	0.9784 (.9782-.9787)	0.9810 (.9808-.9812)	0.8383 (.8378-.8388)	1.148	0.3704 (.3692-.3716)	0.9950 (.9949-.9951)	0.9485 (.9476-.9494)	0.8637 (.8633-.8640)	2.564
Rural area	0.8512 (.8506-.8520)	0.9851 (.9848-.9853)	0.9875 (.9873-.9877)	0.8273 (.8266-.8281)	1.160	0.3406 (.3388-.3425)	1.0000	1.0000	0.8821 (.8816-.8826)	2.947
NCD***										
Yes	0.8166 (.8159-.8174)	0.9860 (.9958-.9862)	0.9808 (.9805-.9811)	0.8595 (.8589-.8600)	1.200	0.3135 (.3117-.3153)	0.9951 (.9950-.9952)	0.9037 (.9017-.9056)	0.9078 (.9074-.9082)	2.844
No	0.8639 (.8636-.8642)	0.9738 (.9736-.9740)	0.9778 (.9777-.9800)	0.8424 (.8420-.8427)	1.130	0.3766 (.3758-.3744)	0.9957 (.9957-9958)	0.9556 (.9551-.9562)	0.8671 (.8669-.8674)	2.558
**Cancer**										
Yes	0.5823 (.5783-.5863)	1.0000	1.0000	0.7964 (.7941-.7987)	1.719	0.3453 (.3399-.3507)	1.0000	0.9478 (.9472-.9483)	0.9241(.9231-.9251)	2.921
No	0.8582 (.8579-.8584)	0.9763 (.9762-.9765)	0.9782 (.9781-.9783)	0.8476 (.8473-.8479)	1.140	0.3674 (.3667-.3682)	0.9954 (.9954-.9955)	1.0000	0.8746 (.8744-.8749)	2.592

*Unweighted number of respondents, Excludes cases for non-respondents of smoking status.

**SN = sensitivity, SP = specificity, PPV = positive predictive value, NPV = negative predictive value, DR = difference ratio (UCC/SRs).

***NCD = Non communicable disease (physician diagnosis about hypertension, diabetes mellitus, dyslipidemia, etc.).

Weights were applied to the SN, SP, PPV, NPV.

**Figure 1:**
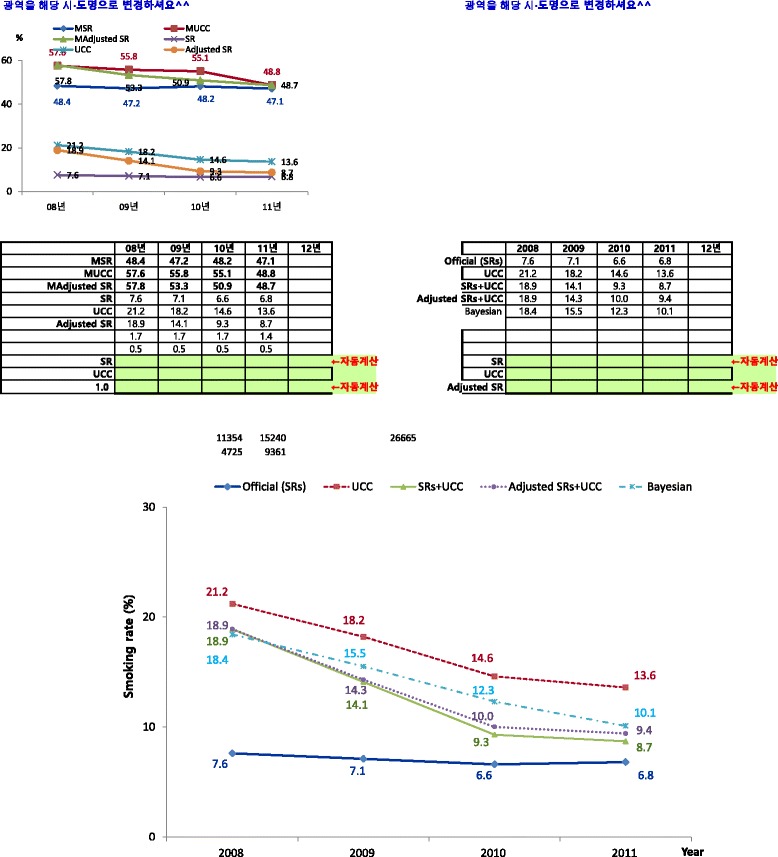
**Female smoking rate by year.** SR: self-reported surveys. UCC: urinary cotinine concentration.

According to the female smoking rate by year, the official smoking rate based on the SRs was 7.6% in 2008, 7.1% in 2009, 6.6% in 2010, and 6.8% in 2011. Conversely, the smoking rate according to the UCC was 21.2% in 2008, 18.2% in 2009, 14.6% in 2010, and 13.6% in 2011. While the difference between the two smoking rates does decrease over time, a difference nevertheless remains. Moreover, if those who responded “no” as to whether they were current smokers on the SRs, but who were considered current smokers according to the UCC, were included in the total number of smokers (SRs + UCC), the female smoking rate would have been 18.9% in 2008, 14.1% in 2009, 9.3% in 2010, and 8.7% in 2011. The UCC study population decreased every year beginning in 2008, with 3,152 participants in 2008, 2,517 participants in 2009, 917 participants in 2010, and 882 participants in 2011. Thus, when the UCC participant number by year was adjusted to analyze each year under the same conditions, the smoking rate (adjusted SRs + UCC) was 14.3% in 2009, 10.0% in 2010, and 9.4% in 2011. The smoking rate according to the Bayesian Theory was 18.4% in 2008, 15.5% in 2009, 12.3% in 2010, and 10.1% in 2011 (see Figure 1).

## Discussion

The present study assesses the response accuracy of SRs regarding smoking status in South Korea using UCC. It is the first study to analyze the annual trends of actual smoking rates in South Korea by reflecting the results of biomarker tests. Significant differences were noted in the smoking rates of both men and women between the SRs and the UCC, showing that many respondents provided false responses. According to conventional studies, the self-reported smoking rates yielded by face-to-face interviews produce values that are lower than the actual smoking rates [5,18]. Although there was a bigger gap between the different measurements of the female smoking rates than those of males, the difference in accuracy between genders was not large [6,19]. However, the accuracy difference between genders is larger than in South Korea; analysis indicates that the difference between the official smoking rate and the actual smoking rate is also very large. Such a result can be attributed to the fact that the social desirability factor has a bigger impact on South Korean women than it does on women in Western countries, as South Korean society often ostracizes and vilifies female smoking. For females living in a minimum two-person household, with either their own family or other people, sensitivity values were low. This may be because female participants did not want to reveal their smoking statuses to family members or neighbors during the KNHANEs household survey. Their low sensitivity values indicate that many females gave false responses to hide the fact that they were smoking. The DR was 2.6, indicating that the actual smoking rate of female respondents was 2.6 times the official smoking rate. On the other hand, the high specificity values of females indicate that occurrences of non-smokers falsely indicating that they do smoke were rare. Also, the NPV value of 0.876 indicates that certain females responded that they were current smokers on the SRs, but the UCC did not detect that these individuals were smokers. One reason for the above result is that the half-life of cotinine is approximately 18 hours [10], so infrequent or light smokers might escape detection [15,20]. The lack of detection can also be attributed to the fact that the UCC threshold for identifying smokers was set too high. If the UCC threshold had been set at a stricter and lower level, the NPV value would rise, and the smoking rate, according to the UCC, would rise. Likewise, when the threshold is eased, the false responses of infrequent or light smokers can be detected. However, setting a high threshold has the disadvantage of increasing false positive responses owing to indirect smoking from exposure to environmental tobacco smoke (ETS) [13,21], food consumption [22], and other daily activities, all of which would increase cotinine levels.

The yearly female smoking rate as officially reported by the government remains consistent at approximately 7%. The official indicator according to SRs steadily decreased from 2008 to 2010 and rose by 0.2% in 2011. However, this phenomenon is not due to an increase in the population of actual smokers but is rather due to the sharp increase in sensitivity levels in 2011. This rise in values indicates that false responses among actual female smokers have decreased. Although there are differences in the UCC smoking rate, the adjusted UCC smoking rate, the SRs + UCC smoking rate, and the adjusted SRs + UCC smoking rate, all of the biological marker indicators exhibit a decreasing trend in smoking.

The strength of the present study is that it calculates South Korea’s actual female smoking rate by using biological indicators. Also, it is the first national study that confirms the changes in smoking rates through a time series analysis. Furthermore, predictions assert that female smoking rates are underestimated to a greater extent in East Asian countries such as South Korea, Japan, and China as well as in Islamic countries as compared to American and European countries, since female smoking is perceived very negatively in the former countries. Consequently, it is necessary to constantly monitor smoking rate trends through cumulative data.

The limitation of the present study is that its calculation of female smoking rates in South Korea cannot be directly generalized to those of other Asian countries. Also, the study could not fully control for the participants the UCC identified as smokers owing to indirect smoking. Furthermore, it is possible that infrequent or light smokers escaped UCC detection as their UCC levels are low.

Unfortunately, KNHANEs no longer performs urinary cotinine tests as of 2012. There are two reasons for this: the government tobacco policy budget decreased, and the fact that hardly any studies utilize this indicator. The present study shows that the accuracy of the smoking rates as reported by SRs was very low, especially among females. Therefore, to accurately gauge smoking rates, it is necessary to reinstitute, consistently study, and utilize the tests in this category.

## Conclusion

Although the official female smoking rate by year remains steady at approximately 7%, this study confirms that the actual female smoking rate is significantly higher than official records state, and it is steadily decreasing. Females exhibited a higher rate of false responses, which results in an underestimation of the female smoking rate. In fact, the smoking rate as indicated by the biological markers was approximately twice as high as the official rate. This discrepancy can be credited to the social desirability bias and the social condemnation of female smoking. It is predicted that East Asian, Southeast Asian, and Islamic countries with similar social cultures regarding female smoking will have more hidden female smokers than Western countries.

### Consent

Interviewers explained the purpose and contents of the survey and examination, provided a informed consent form. And the hand-written signed consent was obtained from all participants.

## Abbreviations

KCDC: The Korea Centers for Disease Control and Prevention
KNHANEs: The Korean National Health and Nutrition Examination Survey
NCD: Non-communicable disease
NPV: Negative predictive value
DR: Difference ratio
PPV: Positive predictive value
SN: Sensitivity
SP: Specificity
SRs: Self-reported surveys
UCC: Urinary cotinine concentration
