# Erratum to: Does South Korea have hidden female smokers: discrepancies in smoking rates between self-reports and urinary cotinine level

**DOI:** 10.1186/s12905-015-0282-2

**Published:** 2016-01-04

**Authors:** Myung Bae Park, Chun-Bae Kim, Eun Woo Nam, Kyeong Soo Hong

**Affiliations:** 1 Department of Health Administration, Yonsei University, Gangwon-Do, Republic of Korea; 2 Department of Preventive Medicine, Yonsei University Wonju College of Medicine, 162 Ilsan-Dong, Wonju-City, Gangwon-Do 220-701 Republic of Korea; 3 Yonsei University Institute for Poverty Alleviation and International Development, Gangwon-Do, Republic of Korea; 4 Healthy City Research Center, Institute of Health and Welfare, Yonsei University, Gangwon-Do, Republic of Korea; 5 Korea Health Promotion Foundation, Seoul, Republic of Korea

Following the publication of this article [1] it was noticed by the author that Fig. 1 contained additional information which should not have been included in the figure. The tables in the figure which were used to create the graph should have been cut from the figure before publication. The correct figure has been included below.

**Fig. 1:**
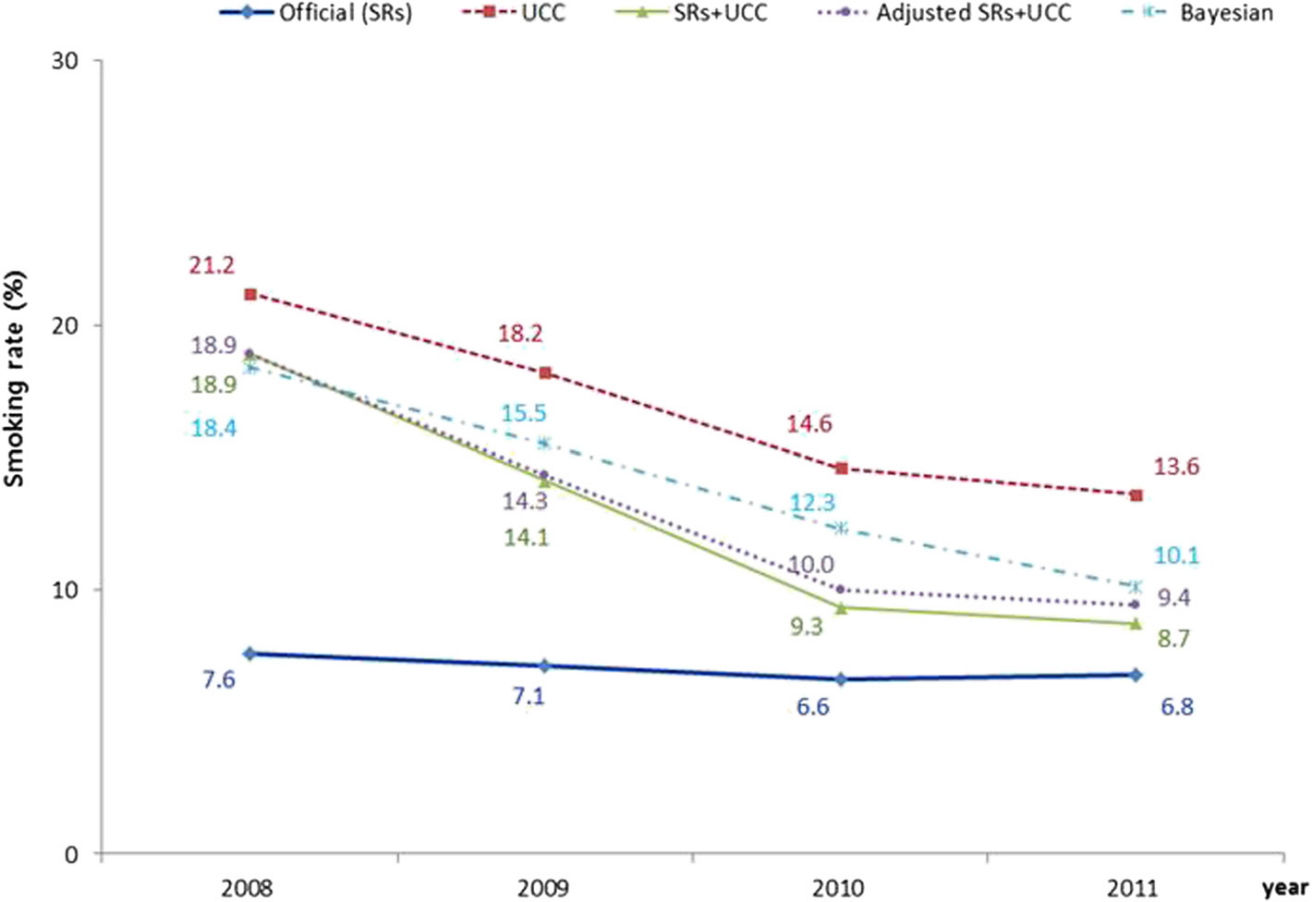
Female smoking rate by year. *SR* self-reported surveys. *UCC* urinary cotinine concentration

## References

[1] Park MB (2014). Does South Korea have hidden female smokers: discrepancies in smoking rates between self-reports and urinary cotinine level. BMC Womens Health.

